# Identification of tumor specimens by DNA analysis in a case of histocytological paraffin tissue block swapping

**DOI:** 10.3325/cmj.2011.52.410

**Published:** 2011-06

**Authors:** Anupuma Raina, Bhuvnesh Yadav, Sher Ali, Tirath Das Dogra

**Affiliations:** 1 DNA Fingerprinting Laboratory, Department of Forensic Medicine and Toxicology, All India Institute of Medical Sciences, Ansari Nagar, New Delhi, India; 2 Molecular Genetics Laboratory, National Institute of Immunology, New Delhi, India

## Abstract

We report on a patient who was diagnosed with high-grade breast carcinoma by all the pre-surgery clinical evidence of malignancy, but histopathological reports did not reveal any such tumor residue in the post-surgical tissue block. This raised a suspicion that either exchange of block, labeling error, or a technical error took place during gross examination of the tissue. The mastectomy residue was unprocurable to sort out the problem. So, two doubtful paraffin blocks were sent for DNA fingerprinting analysis. The partial DNA profiles (8-9/15 loci) were obtained from histocytological blocks. The random matching probability for both the paraffin blocks and the patient’s blood were found to be 1 in 4.43E4, 1.89E6, and 8.83E13, respectively for Asian population. Multiplex short tandem repeat analysis applied in this case determined that the cause of tumor absence was an error in gross examination of the post-surgical tissue. Moreover, the analysis helped in justifying the therapy given to the patient. Thus, with DNA fingerprinting technique, it was concluded that there was no exchange of the blocks between the two patients operated on the same day and the treatment given to the concerned patient was in the right direction.

Short tandem repeats (STR) are reliable markers used for forensic caseworks like human identification and paternity testing (1). These microsatellite markers are being widely used for the identification purpose in mass disaster, rape, and murder cases. Further, they are equally useful to identify mixed biological samples (2). One of the major applications of STR markers is to identify the swapped or contaminated histological paraffin blocks (3,4), which also mostly contain malignant cells. DNA profiling and result interpretation with these samples often becomes a challenging job. The malignant tissues may show preferential amplification, loss of heterozygosity, and microsatellite instability (5,6). Invasive breast cancers, like other malignancies, show microsatellite instability. This subset of breast cancer may affect mono-, di-, and trinucleotide repeats, suggesting a more widespread genomic instability (7). Likewise, loss of heterozygosity is a frequent event in breast cancer (8) but no direct correlation has been observed between microsatellite instability and loss of heterozygosity in breast cancer cases (7,9). There could be a mismatch in the STRs used for the human identification between the normal and cancerous tissue of the same individual due to loss of heterozygosity. Beside all this, DNA STR analysis has proved to be a useful method for resolving formalin-fixed, paraffin-embedded tissue specimens suspected to be misidentified.

Here, we present a case of a 45 years old woman who was diagnosed with breast cancer (invasive ductal carcinoma) by all the screening examinations (breast self-examination, clinical breast examination, and mammography). Definite cancer was revealed from the grade V reporting of Breast Imaging Radiology Data System (BIRADS). Fine needle aspiration cytology and pathological analysis confirmed the cancer. Mastectomy was done with the consent of the patient, despite radiotherapy (as suggested by the treating physician in the hospital). But, in post surgical histopathological examination, no definite residual tumor was seen. As pre-surgery reports showed affirmed tumor (99%), it was thought that either specimen swapping or technical error during tissue block preparation took place. The possibilities were mix-up of specimens in the operating room, mix-up in the pathological laboratory, or a sampling error (no cancerous tissue was examined). As histopathological laboratory of our hospital receives on average 300 or more samples per day, because of shortage of space for specimen storage, the tissue was discarded after histopathological block preparation. Therefore, the experts were not able to reexamine the post-surgical tissue and it was not possible to define the exact reason for the absence of tumor part in the block. The patient believed that she was treated wrongly and she had the full right to press charges. So, it was necessary to explain the actual reason for the negative result of the post-surgical diagnosis. The only way to solve the problem was to match the paraffin block with the patient’s fresh blood to exclude the possibility of sample exchange or a labeling error. On that day, another patient with the same initials was operated on, enhancing the probability of sample mix-up. The other possibilities considered were tissue contamination or the presence of extraneous tissue in paraffin blocks or histological sections, as these are well-recognized problems (up to 8.8%) in surgical pathology. It has been reported that errors related to specimen identification occur in 9.6% of the cases, discrepant or missing information items in 77% of the cases, and specimen handling in 3.6% of the cases (4). Therefore, two tissues fixed in formalin, routinely processed, and embedded in paraffin blocks (15 × 15 × 4mm) of these two patients were sent for DNA analysis, along with the fresh blood of the patient.

## Material and method

DNA was extracted from the fresh blood sample (A) following the organic extraction procedure (10). The two blocks received were labeled as B and C. Due to the difficulty of DNA extraction from the paraffin-embedded blocks, special care was taken during deparaffinization and the existing protocol (11) was modified. Paraffin embedded blocks were first deparaffinized with xylene (twice for 15 minutes) followed by ethanol wash (100%, 75%). The tissues were further washed with phosphate buffered saline and dimethylsulfoxide followed by incubation overnight in 100 μL standard forensic extraction buffer (10 mmol/L Tris, pH 8.0; 50 mmol/L EDTA, pH 8.0; 100 mmol/L NaCl), with 2% sodium dodecyl sulfate and 20 μg/μL of Proteinase K in a thermal shaker at 37°C. DNA extraction was performed using a phenol/chloroform/isoamyl alcohol (25:24:1) organic extraction procedure. The extracted DNA was quantified using Alpha Imager 3400 Imaging System, (Alpha Innotech Corporation, San Leandro, CA, USA) and spectrophotometer (Thermo Spectronic, Rochester, NY, USA). The DNA was purified with the help of centrifugal device (Pall Corporation, Port Washington, NY, USA).

Polymerase chain reaction (PCR) amplification was carried out using the multiplex AmpFlSTR Identifiler PCR amplification kit (Applied Biosystems, Foster City, CA, USA), following the manufacturers’ recommendations. This kit amplifies 15 STR loci and amelogenin in a single reaction. The amplification was carried out in Peltier Thermal Cycler (PTC 200, MJ Research Inc., Waltham, MA, USA) following standard procedures.

Electrophoresis was carried out on an ABI 3130 automated Genetic Analyzer (Applied Biosystems) using 36 cm ID capillary with polymer POP4. One microliter of amplified DNA was mixed with 8.5 μL Hi-Di Formamide (Applied Biosystems, Foster City, CA, USA) and 0.5 μL size standard. Mixture was denatured at 95°C for 5 minutes and chilled on ice. The denatured sample was transferred to autosampler. The data were collected using ABI 3130 data collection software and then analyzed with Genemapper 3.2 software (*http://www.warezdestiny.com/gene-mapper-id-vertion-3.2-download/)* with human identification (HID) application.

## Results

**Figure 1:**
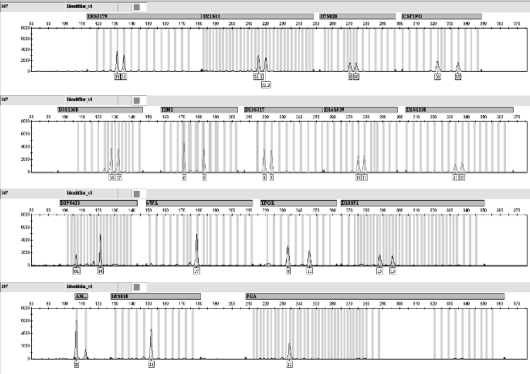
The electropherogram of the patient’s blood sample (Sample A).

**Figure 2:**
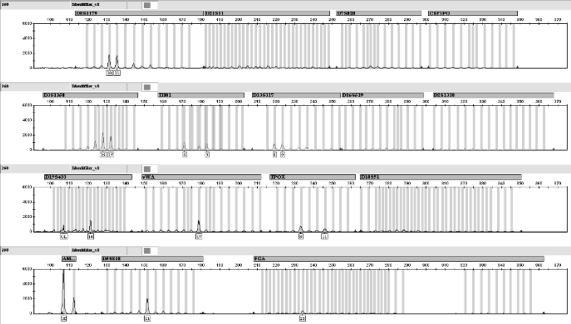
The electropherogram of the histopathological blocked tissue 1 (sample B).

**Figure 3:**
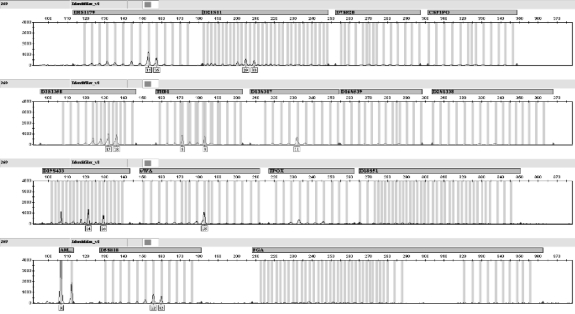
The electropherogram of histopathological blocked tissue 2 (sample C).

In the sample A (Figure 1), all the 15 loci and amelogenin were amplified (Table 1). In the sample B (Figure 2) and C (Figure 3), only 9 and 8 loci, respectively, were amplified. Also, a small peak at Y amelogenin was observed in both blocks, which could be due to the improper sample handling. The sample B showed a complete match with the sample A at the all 9 amplified loci, whereas the sample C showed a match with A only at 1 locus (TH01). The random matching probability of the sample A was 1 in 5.19E15, 8.83E13, and 1.52E14 in Caucasian population, Asian population, and East Indian populations, respectively. The values were sufficiently high to distinguish the individuals. For B (at 9 loci), the values were 1 in 1.57E6, 4.43E4, and 1.36E5 for Caucasian population, Asian population, and East Indian populations, respectively. The random matching probability values for C were 2.56E6, 1.89E6, and 1.27E6 for Caucasian population, Asian population, and East Indian population, respectively.

**Table 1:** The genetic profiles of patient’s blood and both histopathological blocked tissues

	Samples		
Locus	A	B	C
D8S1179	10,11	10,11	15,16
D21S11	31.2,32.2	*	29,30
D7S820	9,10	*	*
CSF1PO	10,13	*	*
D3S1358	16,17	16,17	17,18
THO1	6,9	6,9	6,9
D13S317	8,9	8,9	11,11
D16S539	10,11	*	*
D2S1338	21,22	*	*
D19S433	14,14	14,14	14,16
VWA	17,17	17,17	18,18
TPOX	8,11	8,11	*
D18S51	13,15	*	*
D5S818	11,11	11,11	12,13
FGA	21,21	21,21	*

*No amplification at a particular locus.

## Discussion

In any representative population, repetition of particular 15-loci STR profile is rare because there are many distinct alleles at each of these STR loci. This fact makes it feasible to distinguish between the individuals.

DNA-based PCR tests have proven to be a powerful tool for determining the identity of suspected contaminant tissues or swapping in surgical pathology or cytology cases (12,13). The subsequent description of the human genome has led to the development of new DNA-based methods for identity testing that require minimal amounts of tissue or body fluid for analysis (14). Also, the automated multiplex analysis of loss of heterozygosity and microsatellite instability may be a useful method for cancer screening (both inherited and sporadic) and has a potential prognostic value (15). As fixation of the tissues in formalin and paraffin wax makes remarkable DNA damage, so the complete DNA profiling was not promising. We obtained partial profiles for both the paraffin blocks, but the amplification of 8-9 loci was sufficient to give the conclusive results. No evidence of microsatellite instability or loss of heterozygosity was observed in this case, although the frequency of nuclear microsatellite instability in breast cancer ranges from 0%-80% (16,17) and that of loss of heterozygosity from 30%-60% (18).

The random matching probabilities asserted the minimum probabilities to repeat the same profile even at 9 loci. As sample C was corresponding with A at only 1 locus, the probability of matching of these 2 samples was excluded. As in histopathological laboratory, the sample B was labeled with the patient’s code only, therefore, hypothesis of sample exchange was nullified. Finally, it was concluded that there was neither sample swapping nor a labeling error. The block was correctly labeled and the block without tumor corresponded to the patient only.

This indeed was a case of a technical retrospective error during the gross examination of the post surgical tissue samples and the wrong part was selected for the histopathological analysis. Tissue contamination, absence of correct tissue part, and the presence of extraneous tissue in paraffin blocks, are well-recognized problems in surgical pathology. The frequency of error in pathological reports varies from 0.6% in prospective to 2.9% in retrospective analyses (18). This variation is 0%-8.8% when both prospective and retrospective cases are studied together (19). It often presents a challenge to the pathologist and might result in inappropriate therapy or in additional diagnostic procedures to resolve the uncertainty (20). The use of the STR multiplex system is the method of choice in such situations because of its greater sensitivity and higher discriminating power. Its applicability to detection and determination of the correct samples in surgical pathology was demonstrated in the present study, which helped the patient to receive timely treatment.
